# Development of Positively Charged Poly-L-Lysine Magnetic Nanoparticles as Potential MRI Contrast Agent

**DOI:** 10.3390/nano13121831

**Published:** 2023-06-09

**Authors:** Iryna Antal, Oliver Strbak, Vlasta Zavisova, Jana Vojtova, Martina Kubovcikova, Alena Jurikova, Iryna Khmara, Vladimir Girman, Róbert Džunda, Karol Kovaľ, Martina Koneracka

**Affiliations:** 1 Institute of Experimental Physics, Slovak Academy of Sciences, Watsonova 47, 04001 Kosice, Slovakia; iryna.antal@saske.sk (I.A.); kubovcikova@saske.sk (M.K.); akasard@saske.sk (A.J.); khmara@saske.sk (I.K.); konerack@saske.sk (M.K.); 2 Biomedical Center Martin, Jessenius Faculty of Medicine in Martin, Comenius University in Bratislava, Mala Hora 4, 03601 Martin, Slovakia; jana.vojtova@uniba.sk; 3 Institute of Physics, Faculty of Sciences, Pavol Jozef Safarik University in Kosice, Park Angelinum 9, 04154 Kosice, Slovakia; vladimir.girman@upjs.sk; 4 Institute of Materials Research, Slovak Academy of Sciences, Watsonova 47, 04001 Kosice, Slovakia; rdzunda@saske.sk (R.D.); kkoval@saske.sk (K.K.)

**Keywords:** magnetite nanoparticles, magnetic fluid, superparamagnetic behavior, poly-L-lysine, MRI, relaxometry, relaxation time, relative contrast, relaxivity, enhancement

## Abstract

A colloidal solution of magnetic nanoparticles (MNPs) modified with biocompatible positively charged poly-L-lysine (PLL) with an oleate (OL) layer employed as an initial coating was produced as a potential MRI contrast agent. The effect of various PLL/MNPs’ mass ratios on the samples’ hydrodynamic diameter, zeta potential, and isoelectric point (IEP) was studied by the dynamic light-scattering method. The optimal mass ratio for MNPs’ surface coating was 0.5 (sample PLL_0.5_-OL-MNPs). The average hydrodynamic particle size in the sample of PLL_0.5_-OL-MNPs was 124.4 ± 1.4 nm, and in the PLL-unmodified nanoparticles, it was 60.9 ± 0.2 nm, indicating that the OL-MNPs’ surface became covered by PLL. Next, the typical characteristics of the superparamagnetic behavior were observed in all samples. In addition, the decrease in saturation magnetizations from 66.9 Am^2^/kg for MNPs to 35.9 and 31.6 Am^2^/kg for sample OL-MNPs and PLL_0.5_-OL-MNPs also confirmed successful PLL adsorption. Moreover, we show that both OL-MNPs and PLL_0.5_-OL-MNPs exhibit excellent MRI relaxivity properties and a very high *r*_2_^(^*^)^/r_1_ ratio, which is very desirable in biomedical applications with required MRI contrast enhancement. The PLL coating itself appears to be the crucial factor in enhancing the relaxivity of MNPs in MRI relaxometry.

## 1. Introduction

Iron oxide magnetic nanoparticles (MNPs) consisting of maghemite (γ-Fe_2_O_3_) and/or magnetite (Fe_3_O_4_) particles find applications in magnetic resonance systems, imaging for clinical diagnosis, drug delivery systems, magnetic hyperthermia, etc. [1,2,3,4]. It is well known that bare magnetite nanoparticles are not suitable for biomedical applications due to aggregation. Their surface must be modified to be hydrophilic and biocompatible [5]. The particles must also have limited toxicity, remain uniformly sized under physiological conditions, and effectively accumulate at disease sites [2,6].

In the last two decades, much has been published on wide varieties of coating compounds for magnetic nanoparticles and their use for biomedical applications. Among them, the macromolecule poly-L-lysine (PLL) takes an important place [7,8,9]. PLL is a polymer composed of lysine amino acids that exhibits excellent biocompatibility and biodegradability [10].

Previously, it was demonstrated that poly-L-lysine can coat negatively charged magnetic nanoparticles via electrostatic interaction, which can be successfully used for stem cell labeling [11,12]. H. Khurshid et al. [13] describe heparin-coated MNPs for targeted drug delivery applications in which PLL was used to initially coat these particles to facilitate the subsequent deposition of heparins. The use of PLL-γ-Fe_2_O_3_-NPs as an MRI contrast agent for long-term cell migration studies is described in Ref. [14]. The nanoparticles were prepared by direct flame spray pyrolysis. An oxidative hydrolysis method of MNP preparation followed by coating with PLL was reported by Riggio et al. [15]. Experiments with PLL-Fe_3_O_4_-NPs were performed with the human neuroblastoma SH-SY5Y cell line and primary Schwann cell cultures of the peripheral nervous system.

Earlier studies suggested that poly-L-lysine-modified iron oxide nanoparticles might be a promising gene delivery system that can transfect efficiently in vitro and in vivo [16,17,18].

In our previous paper, we prepared stable PLL-MNP colloids in which PLL was directly adsorbed onto magnetite nanoparticles [19]. Carbonic anhydrase IX (CA IX) specific antibodies were attached to the PLL-modified MNPs. Antibody-coupled PLL-MNPs were used to study the detection and targeting of tumor cells. We showed that PLL-MNPs exhibit cytotoxic activities in a cell-type-dependent manner and bind to cells expressing CA IX when conjugated with the CA IX-specific antibody. Later [20], we confirmed the specific binding of Ab-conjugated PLL-MNPs to CA IX protein in a 3D model of colorectal cancer cells and showed that these nanoparticles could be used for combined magnetic hyperthermia and magnetic resonance imaging.

**Scheme 1:**
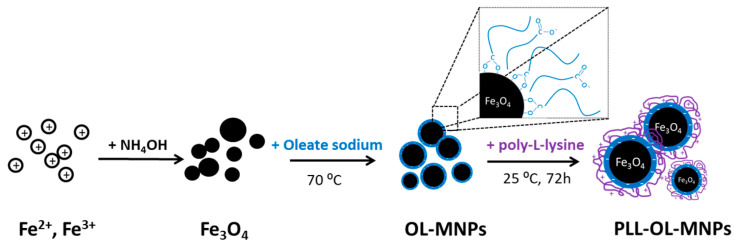
Preparation of PLL-OL-MNPs.

The aim of this study was to prepare positively charged PLL-OL-MNPs in the form of biocompatible magnetic fluid with sodium oleate (OL) employed as an initial coating (Scheme 1). Next, the colloidal stability and aggregation stability of this magnetic fluid in a physiological medium were studied. Finally, the influence of the PLL coating on MRI relaxation properties and MRI contrast using relaxometric protocols were investigated. The high stability of these PLL-OL-MNPs samples under physiological pH conditions and the presence of free -NH_3_^+^ groups on the magnetite surface allow for the conjugation of these nanoparticles with other biomolecules with a variety of therapeutic, targeting, labeling, and MRI agents; therefore, these nanoparticles are promising for a variety of bioapplications.

## 2. Materials and Methods

### 2.1. Materials

Ferric chloride hexahydrate (FeCl_3_·6H_2_O), ferrous sulfate heptahydrate (FeSO_4_·7H_2_O), ammonium hydroxide (NH_4_OH), sodium oleate (C_17_H_33_COONa), poly-L-lysine hydrobromide ((C_6_H_12_N_2_O)_n_, HBr (PLL, Mw = 150–300 kDa)), and Trypan Blue were purchased from Sigma-Aldrich, St. Louis, MO, USA.

### 2.2. Succession of Nanoparticles’ Fabrication

*Synthesis of MNPs.* Magnetite nanoparticles were synthesized by the co-precipitation method. For this purpose, 2.1 g FeCl_3_·6H_2_O and 1.1 g FeSO_4_·7H_2_O were dissolved in 40 mL of distilled water. Under vigorous stirring, 10 mL of 25% ammonium hydroxide solution was added to the flask, and magnetite in the form of a black precipitate was formed. The magnetite was washed three times with 100 mL of distilled water to remove unwanted chemicals (salts) on the particles [21,22,23,24].

*Oleate-coated magnetite nanoparticles (OL-MNPs).* After washing the magnetite by magnetic decantation and subsequently heating it to 80 °C, the surfactant sodium oleate (C_17_H_33_COONa) was added to prevent agglomeration of the particles [25]. The mixture was then stirred and heated until the boiling point was reached. The obtained oleate-bilayer [26]-stabilized magnetite particles were dispersed in water. The agglomerates were removed by centrifugation at 9000 rpm for 30 min [21,25]. The concentration of Fe_3_O_4_ in the sample of OL-MNPs was estimated by thiocyanate colorimetry [27] and was equal to 60 mg mL^−1^. The bilayer-stabilized magnetite nanoparticles prepared by this method were in the form of stable magnetic fluid.

*Poly-L-lysine-modified magnetite nanoparticles (PLL-OL-MNPs).* To determine the optimal conditions for the PLL adsorption on OL-MNPs, a set of samples with a constant concentration of magnetite (0.32 mg mL^−1^) and different amounts of PLL (from 0 to 0.8 mg mL^−1^) was prepared. The set of PLL-OL-MNP samples (mass ratio of R_PLL/Fe3O4_ from 0 to 2.5) was incubated for 72 h at 25 °C. The prepared poly-L-lysine-modified magnetite nanoparticles were stable in the form of magnetic fluid.

### 2.3. Nanoparticles Characterization

Dynamic light scattering (DLS) and zeta potential measurements were performed using Zetasizer Nano ZS equipment (Malvern Instruments, Malvern, UK). DLS measurements of the average particle diameter were performed in the backscattering mode at an angle of 173°. The particle size distribution was characterized by the hydrodynamic diameter, that is, the intensity-weighted mean hydrodynamic diameter of the ensemble collection of particles, as well as by the polydispersity index (PdI) [28] that is a dimensionless measure of the broadness of the size distribution calculated from the cumulants analysis. Zeta potential describing the electrokinetic potential between a colloid’s solid particle and the liquid phase was calculated by measuring the electrophoretic mobilities and then converting collected values by applying the Smoluchowski equation.

UV/Vis spectra of OL-MNPs and PLL_0.5_-OL-MNPs were acquired with a SPECORD^®^ 40 spectrophotometer at wavelengths from 200 to 750 nm using a quartz cuvette.

Thermogravimetric analysis (TGA) was performed to estimate the optimal ratio of PLL/MNPs and confirm PLL binding on the surface of magnetic nanoparticles. Thermal decomposition measurements were performed on dried samples from room temperature up to 900 °C at a heating rate of 15 °C/min in the air using a TGDTA Setaram SETSYS 16 apparatus.

For transmission electron microscopic (TEM) analysis, the samples were diluted in distilled water and ultrasonicated. A droplet of the water-diluted colloidal dispersion was deposited on a copper grid coated with lacey carbon film. The grids were dried in a vacuum. The prepared samples were observed employing a transmission electron microscope (TEM, JEOL JEM 2100F UHR microscope) equipped with a Schottky FEG source and operated at 200 kV acceleration voltage. For imaging of sample nanoparticles, both STEM-BF and TEM-BF modes were used.

Scanning electron microscopy (SEM, JEOL JSM-700F microscope) was used to characterize the morphology of the OL-MNP and PLL_0.5_-OL-MNP samples. Prior to measurements, diluted samples of OL-MNPs and PLL_0.5_-OL-MNPs were dropped on a metal specimen stub, dried under vacuum, and then sputtered with carbon to form a thin carbon layer to prevent charging of the non-conductive samples.

The static response of the sample magnetization to applied magnetic fields, i.e., the *M(H)* curve, was measured at 290 K with a maximum field of 5 T using a commercial Vibrating Sample Magnetometer installed on a cryogen-free superconducting magnet from Cryogenic Limited (London, UK). Before the measurements, the VSM was calibrated using a standard Yttrium iron garnet. The vibration frequency was set to 20.4 Hz. The data were corrected with respect to the magnetization of the empty sample holder.

*Determination of magnetite concentration.* The iron concentration of each colloid was measured by UV/Vis spectrophotometry after the complete dissolution of the MNPs in acid media. Ferrous ions present in the solution were oxidized to ferric ions by H_2_O_2_ prior to reacting with thiocyanate salt to form the iron–thiocyanate complex ([Fe(SCN)_6_]^3−^_(aq)_). Twenty μL aliquots of OL-MNP and PLL_0.5_-OL-MNP samples were completely dissolved in 1 mL of H_2_SO_4(conc)_ for 1.5 h at an elevated temperature (60 °C). Next, 1 mL of 3% H_2_O_2_ was added to each sample, and incubation continued for 30 min at 50 °C. The solutions were transferred to 5 mL volumetric flasks and diluted with distilled water. Then, 1 mL of 1M ammonium thiocyanate solution was added to 1 mL of each sample. After 15 min, the absorbance at 480 nm was measured, and the iron concentration was determined using a SPECORD^®^ 40 spectrophotometer [27].

*Determination of PLL concentration.* The concentration of poly-L-lysine was determined using the method described by Shen [29], whereby Trypan Blue (40 µg mL^−1^) was allowed to react with PLL in sample aliquots diluted in 0.01 M PBS. The amount of PLL adsorbed on MNPs was calculated from a calibration curve constructed for PLL standards from 0 to 12 µg mL^−1^. The real PLL/Fe_3_O_4_ *w*/*w* ratio in the selected (optimal PLL_0.5_-OL-MNPs) sample was 0.325 mg of PLL to 1 mg of Fe_3_O_4_.

### 2.4. Magnetic Resonance Imaging (MRI)

We used three different MRI relaxometric protocols with a Bruker 7 T scanner to determine particle relative contrast (*RC-T*_1_ *weighting*, *RC-T*_2_ *weighting*, *RC-T*_2_* weighting), relaxation times (*T*_1_*, T*_2_*, T*_2_*), and relaxivity values (*r*_1_*, r*_2_*, r*_2_*):

- *T*_1_ mapping—Rapid Acquisition with Refocused Echoes (RARE) pulse sequence, with repetition time *TR* = 5500, 3000, 1500, 800, 400, and 200 ms and echo time *TE* = 7 ms.
- *T*_2_ mapping—Multi-Slice Multi-Echo (MSME) pulse sequence, with repetition time *TR* = 2000 ms, starting echo time *TE* = 8 ms, spacing = 8 ms, and 25 images.
- *T*_2_* mapping—Multi Gradient Echo (MGE) pulse sequence, with a repetition time *TR* = 1200 ms, starting echo time *TE* = 5.1 ms, spacing = 5 ms, and 10 images. Gradient strength: 440 mT/m.

Images were acquired using the following parameters: flip angle (*FA*) = 90°, *FOV* = 8 × 6 cm^2^, matrix = 256 × 192 pixels, and slice thickness = 1 mm. We used the concentration gradient of MNPs (0, 10, 20, 30, 40, 50, 60 µg mL^−1^ of magnetite) to calculate the following MRI parameters.

Relative contrast (*RC*) defines the MRI signal (*S*) change in the presence of MNPs compared to the signal without MNPs (*S*_0_). For MNPs with negative (*S*_0_ > *S*) contrast, properties (iron oxides) are calculated as follows:

(1) $$RC=\frac{\left(S-S_{0}\right)}{S_{0}}$$

Relaxation times (*T*_1_*, T*_2_*, T*_2_*) were determined by fitting the following functions from signal intensity values of different repetition (*TR*) and echo times (*TE*), respectively:

(2) $$M_{z}=M_{0}(1-e^{-TR/T_{1}})$$

(3) $$M_{xy}=M_{0}e^{-TE/T_{2}}$$

where *M_z_* is the z-component of the magnetization vector ***M*** (longitudinal magnetization), *M*_xy_ is the transverse magnetization after the pulse, *M*_0_ is the initial maximum value of magnetization ***M***, TR is the repetition time, *T*_1_ is the longitudinal relaxation time, *TE* is the echo time, and *T*_2_ is the transverse relaxation time. *T*_2_* relaxation time is a combination of the “true” transverse relaxation time *T*_2_ and additional relaxation (*T*_2_′) caused by magnetic inhomogeneities:

(4) $$\frac{1}{T_{2}^{*}}=\frac{1}{T_{2}}+\frac{1}{T_{2}^{\prime }}$$

Relaxivity value *r* (mM^−1^s^−1^) defines the MRI contrast efficiency of MNPs in MRI and is calculated from the slope of inversion relaxation time (defined as relaxation rate *R* (s^−1^)) dependence on iron concentration (mM):

(5) $$R=R_{0}+rC$$

where *R*_0_ is the relaxation rate in the absence of MNPs, *R* is the relaxation rate in the presence of MNPs, and *C* is the iron concentration in the solution of MNPs.

In addition to relaxometry protocols, the susceptibility-weighted protocol (SWI) was used to determine the distortion effect of MNPs on the main magnetic field *B*_0_. The SWI pulse sequence employs the benefits of phase as well as magnitude protocol. The magnetic moment of magnetic nanoparticles interacts with the local magnetic field *B*_0_, altering the phase in the proximity of particles, which results in signal modification.

For MRI data processing, analysis, visualization, and statistics, we used a Paravision “Image Sequence Analysis” tool (Bruker, Billerica, MA, USA) and Matlab R2022b software (Mathworks Inc., Natic, MA, USA)

## 3. Results

MNPs are usually coated with biological molecules to increase the biocompatibility of magnetic nanoparticles. It is known that the direct interaction of lysine residues with a magnetite surface stabilizes the MNPs in solution [11,12,19]. In this work, we prepared positively charged PLL-OL-MNPs in the form of biocompatible magnetic fluid with sodium oleate (OL) employed as an initial coating. We identified the optimal conditions for adsorption polyamino acid PLL on OL-MNPs and characterized their physico-chemical properties. Furthermore, we investigated how the coating of OL-MNPs with PLL affected their MRI relaxation properties and MRI contrast using relaxometric protocols.

### 3.1. Dynamic Light Scattering and Zeta Potential Measurements

Magnetite nanoparticles (MNPs) were synthesized by the standard co-precipitation method [21,22,23,24] followed by coating the MNPs with an oleate double layer [26].

**Figure 1:**
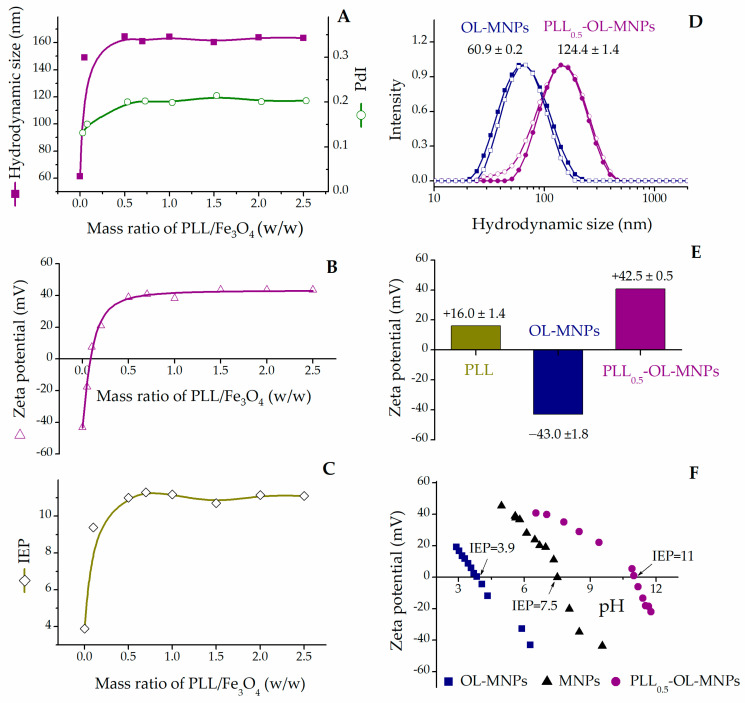
Effects of various theoretical input mass ratios of PLL/Fe_3_O_4_ on the hydrodynamic diameter ((**A**)*,* circles), PdI ((**A**), squares), zeta potential (**B**), and IEP (**C**) of PLL-OL-MNPs. Hydrodynamic particle size distributions of OL-MNP and PLL_0.5_-OL-MNP samples after preparation ((**D**), full figures) and in 6 months ((**D**), empty figures). The zeta potential of pure PLL and OL-MNPs before and after modification by PLL (**E**). pH dependence of the zeta potential of OL-MNPs (*squares*), MNPs (*triangles*), and PLL_0.5_-OL-MNPs (*circles*) (**F**).

The adsorption of PLL onto oleate-coated magnetite nanoparticles was investigated in a set of samples for which the PLL/MNPs’ mass ratio varied from 0 to 2.5 with a constant concentration of magnetite (0.32 mg mL^−1^). The results of the particle size measurements obtained by the DLS method before and after modification with PLL are shown in Figure 1A,B. The hydrodynamic size of the nanoparticles increases with the addition of PLL to the OL-MNP sample until it reaches saturation at a mass ratio of PLL/Fe_3_O_4_ = 0.5. The polydispersity index (PdI) of OL-MNPs and PLL-OL-MNPs was 0.2 (Figure 1A). In the sample PLL_0.5_-OL-MNPs, it can be seen that the adsorption of PLL onto magnetic nanoparticles leads to an increase in their hydrodynamic size by 60 nm compared to PLL-uncoated MNPs (Figure 1A). An additional increase in the PLL/Fe_3_O_4_ mass ratio from 0.5 to 2.5 has no influence on the value of the hydrodynamic size of PLL-OL-MNPs, and at the same time, it does not increase the amount of adsorbed PLL molecules on OL-MNPs’ surface. To reduce the influence of the unbound PLL effect on the sample, the optimal theoretical weight ratio of PLL/Fe_3_O_4_ was determined as 0.5. This is the first point on the saturation zone of the hydrodynamic size measurement curve of obtained DLS data.

The value of the zeta potential indicates the potential stability of nanoparticles in a liquid medium [30]. The dependence of the value of the zeta potential on the PLL/Fe_3_O_4_ ratio is shown in Figure 1B. As seen, even for a fairly small PLL addition (theoretical mass ratio of PLL/Fe_3_O_4_ > 0.2), the particle surface is recharged, and the value of the zeta potential becomes positive until reaching the plateau at PLL/Fe_3_O_4_ from 0.5 to 2.5. The obtained data indicate that the optimal theoretical mass ratio of PLL/Fe_3_O_4_ was 0.5, which is in agreement with the measurement of the hydrodynamic size.

The dependence of the isoelectric point vs. PLL/Fe_3_O_4_ mass ratio was obtained by measuring the zeta potential of the set of PLL-OL-MNP samples (Figure 1C). The saturation begins at the ratio of PLL/Fe_3_O_4_ = 0.5, which agrees well with the optimal ratio from the measurements of the hydrodynamic diameter (Figure 1A) and the zeta potential measurements (Figure 1B). Based on these results, the sample with the optimal theoretical mass ratio of PLL/Fe_3_O_4_ = 0.5 (referred to as PLL_0.5_-OL-MNPs in the text to follow) was used for further investigations.

The isoelectric points (IEP) of pure magnetite, OL-MNPs, and PLL_0.5_-OL-MNPs were determined to confirm poly-L-lysine adsorption onto oleate-modified magnetic nanoparticles. The value of the zeta potential of pure magnetite is gradually reduced from positive in an acidic medium to negative in an alkaline medium crossing zero at pH = 7 [31,32]. According to Figure 1 (1F, triangles), the isoelectric point of pure magnetite was at pH = 7.5. The IEP of the OL-MNPs (Figure 1F, blue line) was approximately 4. The shift of IEP to lower pH values indicates the coating of nanoparticles with sodium oleate [30,33]. The subsequent modification of the OL-MNPs with poly-L-lysine is accompanied by a gradual shift of the IEP to the alkaline medium (Figure 1F, violet line) in the direction of the IEP of pure PLL, whose value in the literature is in the range from 9.47 to 10.6 [31,34,35]. The positive charge values of the PLL-OL-MNPs compared to OL-MNPs as well as the shift of the isoelectric point are caused by the presence of the PLL amino groups, which confirms the successful amino functionalization of the OL-MNPs’ surface.

The sample of PLL_0.5_-OL-MNPs’ colloid was stable to aggregation for over six months after its preparation (Figure 1D). The hydrodynamic size of OL-MNPs and PLL_0.5_-OL-MNPs was practically unchanged over time. There was no gradual decrease in the zeta potential values with the storage time (not shown). These outcomes indicate that no particle agglomeration or aggregation occurred in the suspension during the entire period.

Figure 1E shows the surface charge (zeta potential) of the nanoparticles in OL-MNPs before and after coating with PLL. The PLL itself has a zeta potential of +16 mV. The zeta potentials of the OL-MNPs and PLL_0.5_-OL-MNPs are −43.0 ± 1.8 and 42.5 ± 0.5 mV, respectively, which confirms modification of the surface of the oleate nanoparticle by PLL. Furthermore, the absolute values of the zeta potential are greater than +40 mV, indicating that the suspensions of OL-MNPs and PLL_0.5_-OL-MNPs are stable [10,36].

Therefore, it can be concluded that the amino groups of PLL on the surface of oleate-modified magnetic nanoparticles are responsible for the positive value of the zeta potential of PLL_0.5_-OL-MNP sample, and the mass ratio of PLL/Fe_3_O_4_ = 0.5 (sample PLL_0.5_-OL-MNPs) is optimal for the formation of the PLL shell.

### 3.2. Thermogravimetric Analysis of OL-MNPs and PLL-OL-MNPs

**Figure 2:**
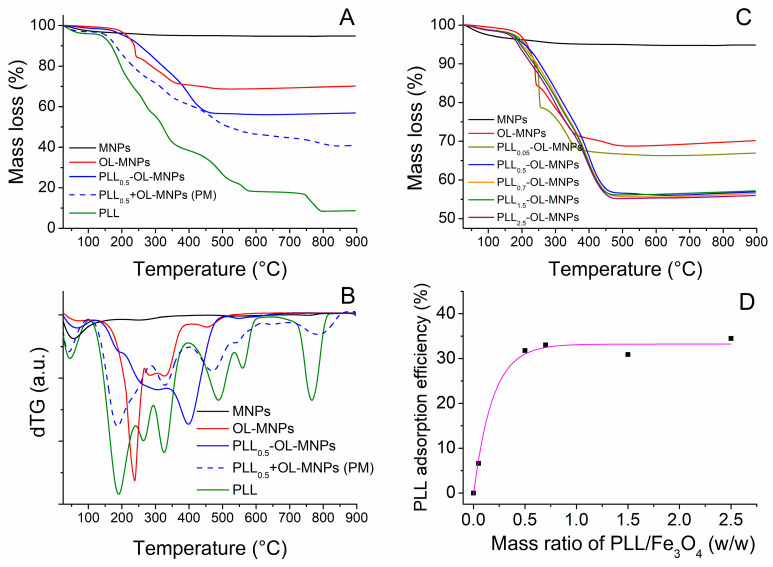
TG and derivative dTG curves for the samples of bare nanoparticles, nanoparticles coated with sodium oleate, pure PLL, nanoparticles functionalized with PLL, and physical mixture (PM) of PLL and oleate-coated nanoparticles for input PLL/MNPs = 0.5 (*w*/*w*) (**A**,**B**). Thermograms of MNPs, OL-MNPs, and the samples with different PLL/MNP input ratios (**C**). Dependence of PLL adsorption efficiency on the input ratio of PLL/Fe_3_O_4_ (*w*/*w*) (**D**).

Thermogravimetric (TG) measurements were performed to confirm the successful coating of OL-MNPs by PLL, and the representative results are shown in Figure 2. The confirmation of PLL bounding on OL-MNPs is revealed in Figure 2A,B, where TG and derivative TG curves of pure PLL, bare magnetic nanoparticles, MNPs coated with sodium oleate, OL-MNPs functionalized with poly-L-lysine, and a physical mixture of OL-MNPs with PLL with the input *w*/*w* PLL/Fe_3_O_4_ ratio of 0.5 are illustrated. Adsorbed water evaporation is observed up to 100 °C for all the investigated samples. This is followed by several stages of decomposition up to a temperature of 900 °C, depending on the sample. Although the decomposition of PLL in the physical mixture of PLL and MNPs is a superposition of the decomposition stages of pure PLL and OL-MNPs, the decomposition of PLL in PLL-bounded MNPs occurs at a lower temperature.

Figure 2C shows the TG thermograms for the samples of PLL-functionalized magnetic nanoparticles with different input PLL/Fe_3_O_4_ ratios from 0.05 to 2.5 and for the bare MNPs as well as the OL-MNPs. Only about 5% mass loss was detected for the pure magnetic nanoparticles due to water evaporation. For OL-MNPs, there was a total mass loss of approximately 30% over the temperature range up to 500 °C; therefore, about 25% of sodium oleate was bounded on MNPs. The mass loss of PLL-functionalized samples is from 3 to 14%, depending on the input PLL/Fe_3_O_4_ ratio. To determine the optimal input ratio of PLL covering the magnetic nanoparticle surface, the adsorption efficiency was obtained from the TG residuals at the temperature of 850 °C. The effect of different input PLL ratios on PLL adsorption efficiency calculated as a ratio of the actual adsorbed amount of PLL to input loading PLL expressed in percentage is displayed in Figure 2D. As can be seen, the saturation starts at a PLL/Fe_3_O_4_ ratio of about 0.6 *w*/*w,* indicating that a maximum of 33.2% of the input PLL amount, i.e., 0.332 mg PLL/mg Fe_3_O_4_ can be bounded onto MNPs. The result agrees with the UV/Vis measurement value, which corresponds to the dosage of 0.325 mg PLL/mg Fe_3_O_4_ (see Table 1) [29].

### 3.3. Spectroscopic Characterization of OL-MNPs and PLL_0.5_-OL-MNPs

**Figure 3:**
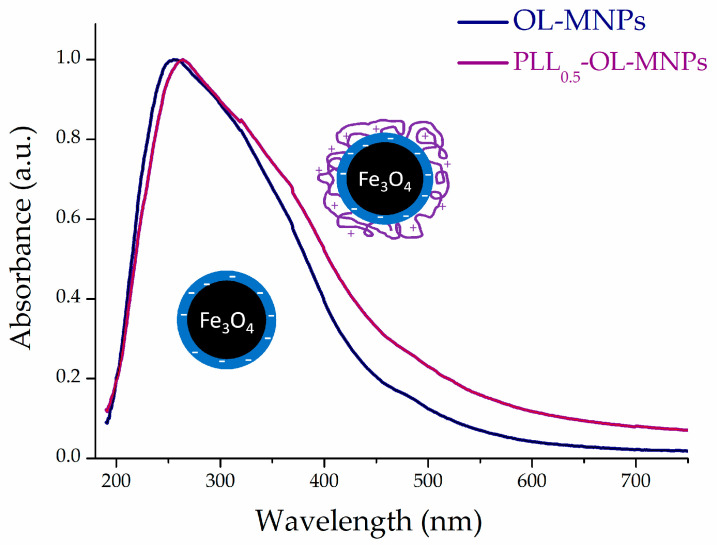
UV/Vis spectra of OL-MNPs (blue) and PLL_0.5_-OL-MNPs (violet).

Other manifestations of the interaction were observed by UV/Vis absorption spectroscopy (Figure 3). A spectroscopic experiment was used to confirm the adsorption of PLL on oleate-modified magnetite nanoparticles. The OL-MNPs have an absorption maximum at 255 nm, which shifts to 264 nm after the adsorption of PLL onto the oleate layer of nanoparticles [10]. UV/Vis proves that PLL forms a polymer layer around the magnetite nanoparticles coated with sodium oleate without inducing their aggregation.

### 3.4. TEM and SEM Analysis

**Figure 4:**
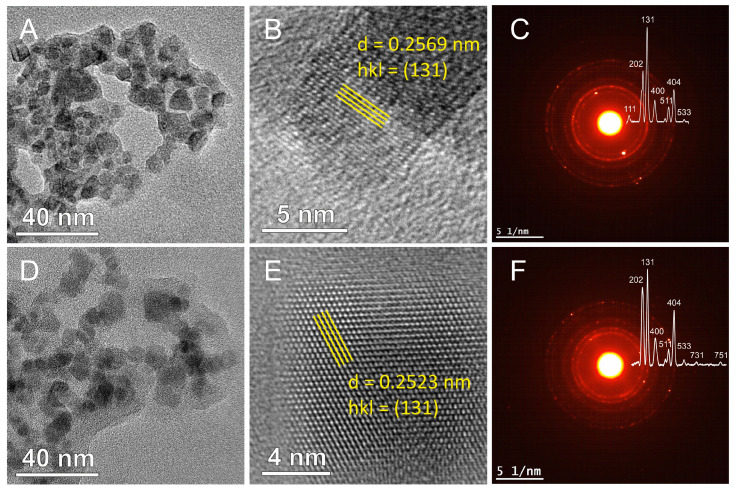
TEM and HRTEM images of OL-MNPs (**A**–**C**) and PLL_0.5_-OL-MNPs (**D**–**F**) and the corresponding diffractions patterns.

TEM and HRTEM images were also acquired to assess the surface morphology of OL-MNPs’ and PLL_0.5_-OL-MNPs’ nanostructures (Figure 4). According to HRTEM images, the synthesized nanoparticles were quasi-spherical with an average size of 7–9 nm (Figure 4A,D). Measured interplanar distances in HRTEM images were 0.2569 nm (Figure 4B) and 0.2523 nm (Figure 4E). The same distances were found in reciprocal space represented by electron diffraction patterns. Distances correspond to the (131) lattice plane system of the Fe_3_O_4_ phase (SG: 227, Fd-3m, cubic crystal system). The Fe_3_O_4_ phase was confirmed by analysis of all diffraction rings. Before processing, the electron diffraction patterns were radially integrated. The obtained diffraction profiles are embedded in diffraction patterns (Figure 4C,F). The mean size of the magnetic cores of the MNPs, OL-MNPs, and PLL_0.5_-OL-MNPs obtained from the analysis of TEM images is in good agreement with the size of the magnetic cores obtained from magnetic measurements (see Table 1).

**Figure 5:**
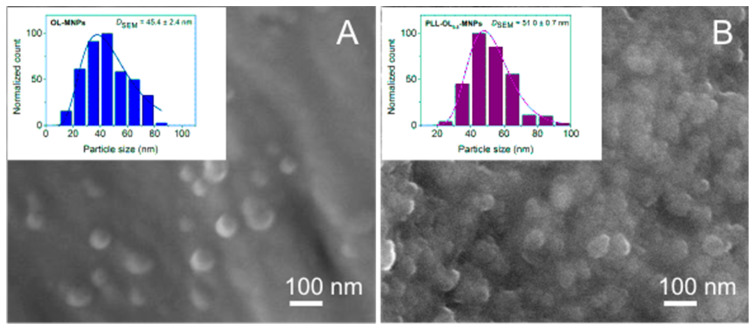
SEM image of nanoparticles in the samples of OL-MNPs (**A**) and PLL_0.5_-OL-MNPs (**B**) with corresponding size distributions (insets).

SEM images were recorded to visualize the surface morphology of OL-MNPs and PLL_0.5_-OL-MNPs (Figure 5). These images reveal that the OL and PLL coatings provided nanoparticles with a smooth surface. Both samples, the OL-MNPs and PLL_0.5_-OL-MNPs, exhibited a uniform spherical structure; however, the size of functionalized PLL_0.5_-OL-MNPs particles is relatively larger. The size distributions constructed based on SEM image evaluation are shown in the insets of Figure 5. The solid lines correspond to the lognormal distribution fit and give us an average particle size of OL-MNPs and PLL_0.5_-OL-MNPs equal to 45.4 and 51.0 nm, respectively.

Table 1 summarizes the results of the OL-MNPs’ and PLL0.5-OL-MNPs’ particle sizes obtained by SEM and DLS measurements. It was expected that the values from DLS (intensity-weighted mean hydrodynamic diameter) would be slightly higher than those from SEM due to the fact that the DLS method, based on the Brownian motion of dispersed particles in a liquid, measures the hydrodynamic diameter and also takes into account the thickness of the liquid layer on the particle surface that moves along with it [37]. The larger dimension obtained by the DLS method can also be explained by the fact that the particle size distribution is not narrow, and thus the presence of larger particles can contribute to the increase in light scattering, thus shifting the measured particle size towards larger values.

### 3.5. Magnetic Properties of OL-MNPs and PLL_0.5_-OL-MNPs

**Figure 6:**
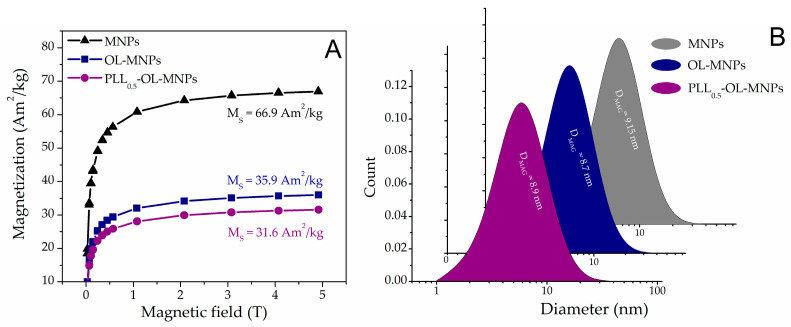
Magnetization curves (**A**) and magnetite core size distributions (**B**) of MNP, OL-MNP, and PLL_0.5_-OL-MNP samples.

Magnetization measurements were performed to confirm the prepared samples’ superparamagnetic behavior and to evaluate the effect of functionalization on the magnetic properties of MNPs. In all measured samples, superparamagnetic behavior was confirmed (data are not shown). The samples did not exhibit hysteresis, and neither coercivity nor remanence was detected. The saturation magnetizations of the samples were found to be 66.9, 35.9, and 31.6 Am^2^/kg for the samples of uncoated MNPs, OL-MNPs, and PLL_0.5_-OL-MNPs, respectively (Figure 6A). As seen in the figure, a decrease in magnetization with increasing coating layers is observed. This is because magnetization is proportional to the amount of mass for the same magnetic material. Increasing the coating layer increases the amount of nonmagnetic material on iron oxide. This means that the greater the layer of the coating, the less magnetite contained in the same mass of the sample. We fitted magnetization curves by the Langevin function [38] and obtained the magnetic core diameter distribution (Figure 6B). As seen in the figure, the magnetite core diameter was approximately the same in all measured samples, resulting in the conclusion that the functionalization of magnetic nanoparticles does not influence the magnetite core.

In addition, the values of saturation magnetization were also used to estimate the magnetite content in the samples by dividing the saturation magnetization of the coated samples by the saturation magnetization of pure magnetic nanoparticles. Subsequently, the amount of PLL adsorbed on the MNP surface was calculated. The value is a little lower than the corresponding results of the TGA and UV/Vis analysis (see Table 1).

### 3.6. Aggregation and Colloidal Stability of PLL_0.5_-OL-MNPs vs. Temperature and Ionic Strength of the Solution

Aggregation and colloidal stability of OL-MNPs’ and PLL_0.5_-OL-MNPs’ oxide nanoparticles were systematically examined as a function of the temperature and ionic strength of the solution using the DLS technique.

**Figure 7:**
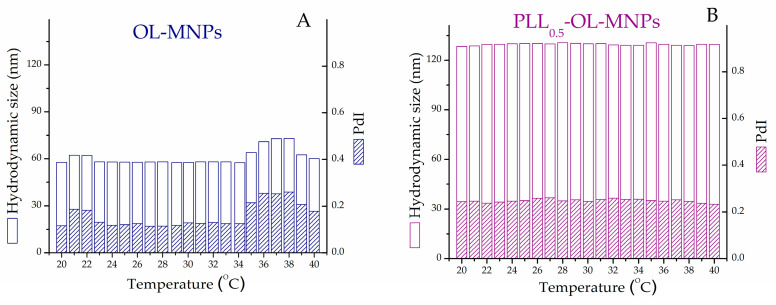
Aggregation stability of the OL-MNP (**A**) and PLL_0.5_-OL-MNP (**B**) samples vs. temperature.

Figure 7 illustrates the variation in the hydrodynamic size of magnetite nanoparticles coated with oleate (A) and functionalized with poly-L-lysine (B) as a function of temperature. DLS measurements were carried out at temperatures ranging from 20 to 40 °C at 1 °C step and with temperature stabilization for a period of 3 min before each measurement. The hydrodynamic size of the particles did not change between 20 and 40 °C. Similar behavior was observed for both OL-MNPs and PLL_0.5_-OL-MNPs.

**Figure 8:**
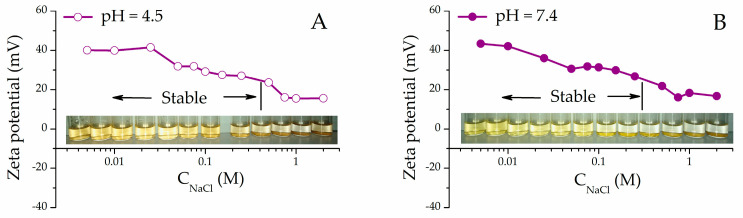
Ζeta potential of PLL_0.5_-OL-MNPs as a function of the ionic strength at pH of 4.5 (**A**) and 7.4 (**B**).

The colloidal stability of PLL_0.5_-OL-MNPs vs. ionic strength was studied by DLS measurements. Generally, increasing the ionic strength of the electrolyte solution compresses the diffused layer associated with NPs, resulting in lower zeta potential on the surface and diminished repulsion between NPs, promoting particle aggregation [39,40]. The surface charge (zeta potential) of the nanoparticles in the PLL_0.5_-OL-MNP sample at a pH of 4.5 (Figure 8A) and 7.4 (Figure 8B) in an electrolyte (NaCl) solution was measured to study the effect of ionic strength on the aggregation of MNPs. Different NaCl concentrations (from 0.05 to 2 M) were added to the solution of poly-L-lysine-coated nanoparticles. In the PLL_0.5_-OL-MNP sample, the zeta potential value is more than +25 mV up to 0.3 and 0.2 M NaCl in solutions with a pH of 4.5 and 7.4, respectively. Then, the zeta potential decreased with an increasing concentration of NaCl in the solution. The results of this experiment show that the prepared poly-L-lysine-modified nanoparticles are suitable for further biomedical applications due to their high positive effective surface charge and good colloidal stability under physiological pH conditions (C_NaCl_ = 0.145 mM).

### 3.7. Magnetic Resonance Imaging

**Figure 9:**
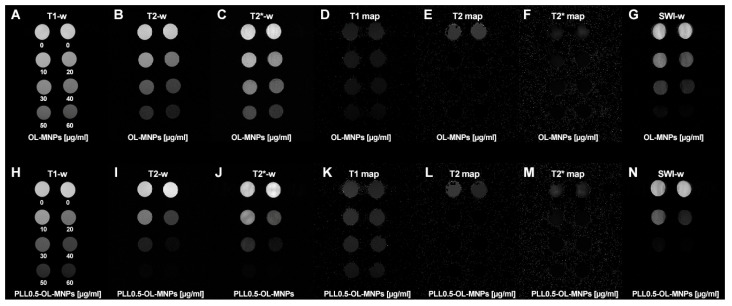
MRI images of OL-MNPs and PLL_0.5_-OL-MNPs acquired by different weighting protocols: (**A**) *T*_1_-weighted, (**B**) *T*_2_-weighted, (**C**) *T*_2_*-weighted, (**D**) *T*_1_ map, (**E**) *T*_2_ map, (**F**) *T*_2_* map, (**G**) SWI-weighted OL-MNPs, (**H**) *T*_1_-weighted, (**I**) *T*_2_-weighted, (**J**) *T*_2_*-weighted, (**K**) *T*_1_ map, (**L**) *T*_2_ map, (**M**) *T*_2_* map, (**N**) SWI-weighted PLL_0.5_-OL-MNPs. The concentration gradient of MNPs from top to bottom, left to right: 0 μg mL^−1^ MNPs (redistilled water), 0, 10, 20, 30, 40, 50, 60 μg mL^−1^ MNPs (OL/PLL_0.5_-OL).

Finally, OL-MNPs and PLL_0.5_-OL-MNPs were investigated by MRI techniques to reveal the relaxation and contrast properties of MNPs coated with OL and PLL. A comparison of images of OL-MNPs and PLL_0.5_-OL-MNPs with different weighting and relaxation time maps is shown in Figure 9. Even with the naked eye, a significant shortening of the signal intensity (hypointensity) in PLL_0.5_-OL-MNPs compared to OL-MNPs, especially for higher concentrations of MNPs, is visible for all weighted pulse sequences: *T*_1_-weighted (Figure 9, A vs. H), *T*_2_-weighted (Figure 9, B vs. I), *T*_2_*-weighted (Figure 9, C vs. J), and susceptibility-weighted (Figure 9, G vs. N). Neither OL nor PLL themselves in concentrations used in our MRI experiments possess magnetic properties that could affect the MRI signal (unpublished results + [20]).

**Figure 10:**
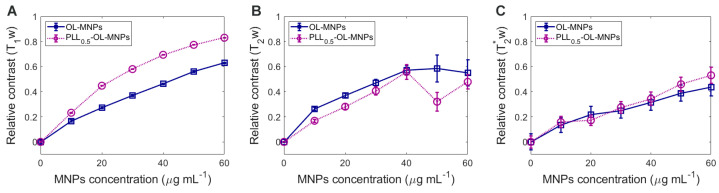
Relative contrast of OL-MNPs and PLL_0.5_-OL-MNPs acquired by different weighting protocols: (**A**) *T*_1_-weighted RARE, (**B**) *T*_2_-weighted MSME, (**C**) *T*_2_*-weighted MGE.

**Figure 11:**
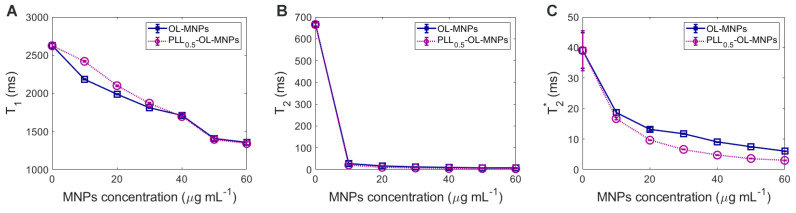
Relaxation times of OL-MNPs and PLL_0.5_-OL-MNPs acquired by different weighting protocols: (**A**) *T*_1_-weighted RARE, (**B**) *T*_2_-weighted MSME, (**C**) *T*_2_*-weighted MGE.

However, except for the *T*_1_-weighted protocol, the relative contrast data are not so pronounced (Figure 10). This points to increased spin–lattice (longitudinal) interaction, most likely caused by PLL’s molecular weight. The size of PLL molecules is also the reason (a mechanical barrier) for a minimal spin–spin (transverse) interaction, as shown in Figure 10B,C. This is also confirmed by the graphs of the relaxation times shown in Figure 11.

**Figure 12:**
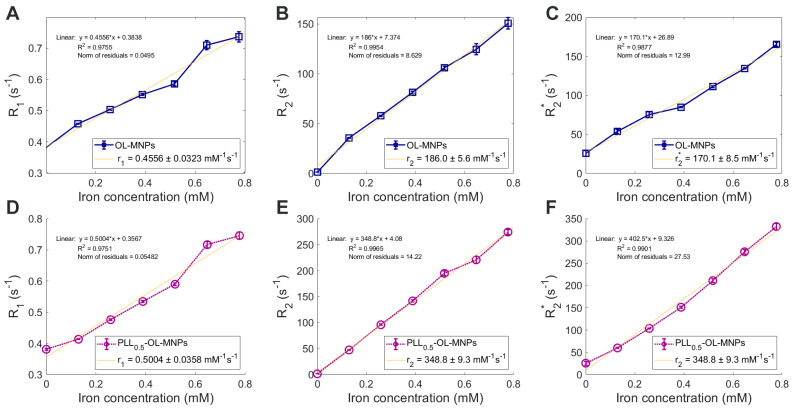
Determination of relaxivities from the slope of relaxation rate’s dependence on iron concentration: (**A**) *r*_1_, (**B**) *r*_2_, (**C**) *r*_2_* for OL-MNPs and (**D**) *r*_1_, (**E**) *r*_2_, (**F**) *r*_2_* for PLL_0.5_-OL-MNPs.

**Figure 13:**
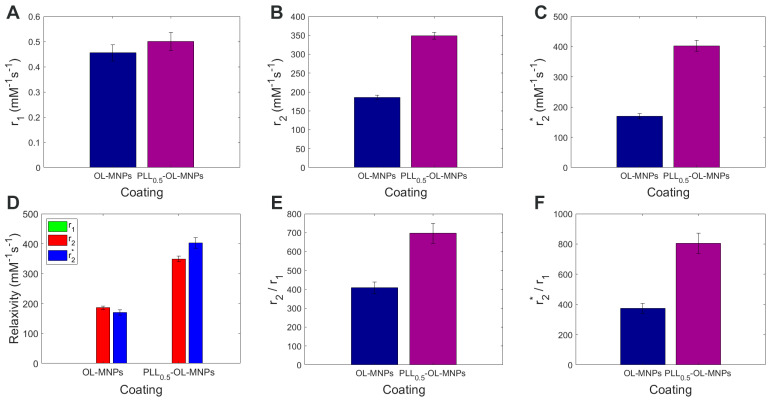
Comparison of relaxivities of OL-MNPs and PLL_0.5_-OL-MNPs: (**A**) *r*_1_, (**B**) *r*_2_, (**C**) *r*_2_*, (**D**) *r*_1_ vs. *r*_2_ vs. *r*_2_*, (**E**) ratio *r*_2_*/r*_1_, (**F**) ratio *r*_2_*/r_1_.

**Figure 14:**
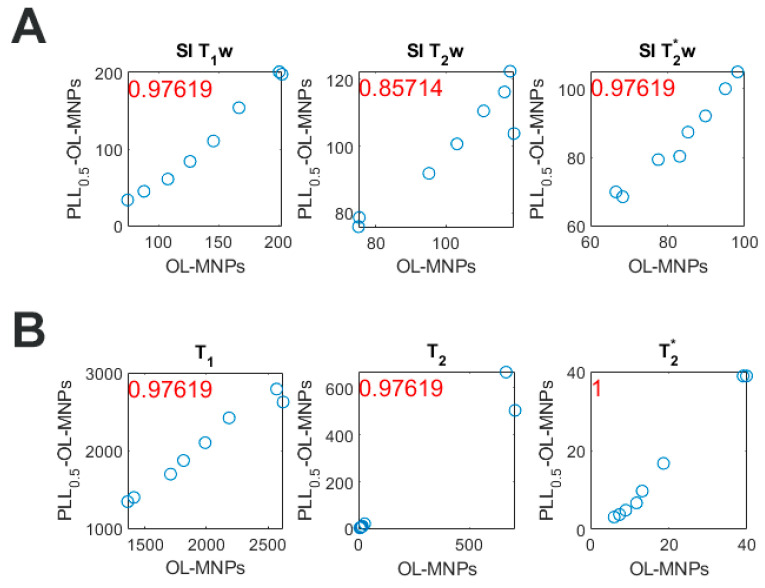
Spearman’s correlation coefficients *(CC)* of OL-MNPs and PLL_0.5_-OL-MNPs: (**A**) signal intensity *(SI) CC*, (**B**) relaxation time *T CC*.

Despite ambiguity in the relative contrast and relaxation time values, the calculated relaxivities (Figure 12) of OL-MNPs and PLL_0.5_-OL-MNPs differ significantly in transverse relaxivity *r*_2_^(^*^)^ value(s) (Figure 13B,C) compared to the longitudinal relaxivity *r*_1_ (Figure 13A). Further, since the relaxivity *r* is the main quantity that defines MRI relaxation properties of the paramagnetic substance, we consider the results in Figure 13D authoritative, whereby the longitudinal relaxivity value r_1_ is so small compared to transverse relaxivities *r*_2_ and *r*_2_* that it is invisible (on the order of 1000 times less). Moreover, the transverse to longitudinal relaxivity ratio (*r*_2_^(^*^)^/r_1_) is used as a key tool in comparing MRI contrast properties of various compounds. Figure 13E,F shows that both ratios significantly differ between OL-MNPs and PLL_0.5_-OL-MNPs and prove the prevailing transverse relaxivity in the systems of OL-MNPs and PLL_0.5_-OL-MNPs. This study’s primary goal was to determine the relaxation properties and MRI differentiation between the OL-MNPs and PLL_0.5_-OL-MNPs. Although PLL possesses no ferro- or ferrimagnetic properties, we confirmed that PLL coating affects the relaxivity properties of MNPs significantly. The main reason for this is most likely the shielding effect of large PLL molecules on MNPs, which partially affects spin–spin and spin–lattice interactions (Figure 10 and Figure 11) but does not alter the overall magnetic properties of OL-MNPs’ and PLL_0.5_-OL-MNPs’ systems (Figure 13). This is also supported by the high values (very strong positive correlation) of the Spearman correlation coefficients (*CC*) shown in Figure 14. Spearman *CC* quantifies the degree of linear association between two parameters where a normal distribution is not required. In all analyzed parameters, the increase in the value of OL-MNPs is associated with a similar increase in the value of PLL_0.5_-OL-MNPs, which would not be possible if the PLL affected the magnetic properties of MNPs, indirectly proving the non ferro- or ferrimagnetic properties of the PLL.

In addition, we compared the relaxivity values of the OL-MNP and PLL_0.5_-OL-MNP systems with our previous studies related to the different coatings of MNPs and commercially available MNPs based on iron oxides and used as MRI contrast agents [20,22,41,42,43,44]. As seen in Table 2, the current system of OL-MNPs and PLL_0.5_-OL-MNPs exhibits excellent *r*_2_^(^*^)^/*r*_1_ ratio values compared to other MNPs. For the PLL_0.5_-OL-MNP system, the values are twice as large as those that we have measured so far for chitosan-stabilized MNPs (Chit-MNPs) [22]. This proves the significantly prevailing transverse relaxivity in MNPs based on iron oxides and stabilized with PLL. In addition, it seems that the presence of OL decreases both relaxivities in PLL-stabilized MNPs (compare PLL-MNPs vs. PLL_0.5_-OL-MNPs in Table 2), which points to the fact that PLL coating is the crucial factor in the enhanced MRI relaxivity of MNPs, as previously shown [20].

## 4. Conclusions

A simple methodology to synthesize poly-L-lysine-modified magnetite nanoparticles was developed in which an oleate layer was employed as an initial coating of magnetite nanoparticles. The prepared sample of PLL_0.5_-OL-MNPs has appropriate characteristics, mainly from the point of view of size, charge, magnetic properties, and stability, for utilization in the field of bioapplication. Magnetic nanoparticles are superparamagnetic with relatively high saturation magnetization, with good stability at a neutral pH even after six months of the sample preparation. The PLL functionalization of OL-MNPs increases the hydrodynamic size due to the presence of a protective layer on the surface of the nanoparticles. In addition, compared to other MNPs, both systems of OL-MNPs and PLL_0.5_-OL-MNPs exhibit excellent transverse relaxivity properties and a very high *r*_2_^(^*^)^/r_1_ ratio, which is very desirable in practical applications. It seems that the PLL coating itself significantly increases the longitudinal and transverse relaxivity of MNPs based on iron oxides and can be used in biomedical applications with required MRI contrast enhancement. However, before their use in practical applications, the stability and toxicity of OL-MNPs and PLL_0.5_-OL-MNPs in vivo must be evaluated, which is our next planned research step in studying positively charged poly-L-Lysine magnetic nanoparticles as potential MRI contrast agents.

## Figures and Tables

**Table 1:** Properties of MNPs, OL-MNPs, and PLL_0.5_-OL-MNPs.

Sample	Diameter (nm)			PdI	Zeta Potential (mV)	*M*_s_ (Am^2^/kg MNPs)	Adsorbed PLL onto MNPs (mg/mg)		
	*D*_SEM_	*D*_MAG_	*D*_DLS_				UV/Vis	TGA	Mag. Meas.
MNPs	-	9.15	-	-	-	66.9	-	-	-
OL-MNPs	45.4	8.7	60.9 ± 0.2	0.118 ± 0.007	−43.0 ± 1.8	35.9	-	-	-
PLL_0.5_-OL-MNPs	51.0	8.9	124.4 ± 1.4	0.244 ± 0.011	+42.5 ± 0.5	31.6	0.325	0.332	0.251

*D*_SEM_ (nm)—the mean size of magnetic particles evaluated from SEM images; *D*_MAG_ (nm)—magnetic core diameter obtained from Langevin function; *D*_DLS_ (nm)—z-average of magnetic particle size measured by DLS measurements; PdI—polydispersity index obtained by DLS measurements; zeta potential (mV)—zeta potential of the samples; *M*s (Am^2^/kg)—saturation magnetization of the samples; adsorbed PLL onto MNPs (mg/mg)—the amount of PLL adsorbed on MNPs (mg/mg) determined by UV-Vis spectroscopy, TGA, and magnetic measurements.

**Table 2:** Comparison of relaxivity values and relaxivity ratios of OL-MNP and PLL_0.5_-OL-MNP systems (green) with MNPs with different coating from our previous studies (gray) and commercially available MNPs based on iron oxides and used as MRI contrast agents (blue).

Magnetic Nanoparticles	*r*_1_ (mM^−1^s^−s^)	*r*_2_ (mM^−1^s^−s^)	*r*_2_* (mM^−1^s^−s^)	*r*_2_/r_1_	*r*_2_*/r_1_	*B*_0_ (T)	References
Resovist	2.8	176	NA	63	NA	4.7	[41]
Feridex	2.3	105	NA	46	NA	4.7	[41]
Feraheme	3.1	68	NA	22	NA	7	[42]
ZES-SPIONs	3.4	60	NA	18	NA	7	[42]
MNPs	2.4	201.9	285.5	84	119	7	[43]
Chit-MNPs	0.7	238.2	276.1	**340**	**394**	7	[22]
PLL-MNPs	1.8	**487.9**	**604.7**	271	336	7	[20]
Lys-MNPs	2.4	199.6	201.8	83	84	7	[43]
Gly-MNPs	2.4	192.6	225.1	80	94	7	[43]
Trp-MNPs	0.9	67.6	256.3	75	285	7	[43]
Dex^40^-MNPs	2.2	198.1	NA	90	NA	7	[44]
Dex^70^-MNPs	1.7	221.5	NA	130	NA	7	[44]
Dex^150^-MNPs	2.0	297.1	NA	149	NA	7	[44]
OL-MNPs	0.5	186.0	170.1	**372**	**340.2**	7	[This work]
PLL_0.5_-OL-MNPs	0.5	**348.8**	**402.5**	**697.6**	**805**	7	[This work]

Chit—chitosan, PLL—poly-L-lysine, Lys—lysine, Gly—glycine, Trp—tryptophan, Dex—dextran (with different molecular weights).

## Data Availability

The data that support the findings of this study are available on request from the corresponding author.
